# Parallel Mediating Roles of Illness Acceptance Attitudes Between Physical Symptoms and Quality of Life in Patients with Gastrointestinal Cancers

**DOI:** 10.1007/s11596-026-00186-9

**Published:** 2026-04-02

**Authors:** Yue-jiao Fan, Jian-li Hu

**Affiliations:** Cancer Center, Union Hospital, Tongji Medical College, Huazhong University of Science and Technology, Wuhan, 430022 China

**Keywords:** Gastrointestinal cancer, Illness acceptance, Quality of life, Physical symptoms, Mediation analysis, Psychological adjustment

## Abstract

**Objective:**

Physical symptoms severely impair quality of life (QoL) in patients with gastrointestinal cancers (GICs). Although illness acceptance is recognized as a key mediator, the distinct rols of its two dimensions—active acceptance and negative acceptance—remain underexplored in the symptom-QoL pathway. This study aimed to explore their parallel mediating roles.

**Methods:**

In this cross-sectional study, 301 inpatients with GICs completed the MD Anderson Symptom Inventory (MDASI), the 12-item Short Form Health Survey (SF-12), the Illness Cognition Questionnaire-Acceptance subscale (ICQ-A, measuring active acceptance), and the Medical Coping Modes Questionnaire (MCMQ, measuring negative acceptance). Parallel mediation analysis was performed using PROCESS Model 4 with bootstrap resampling (5,000 iterations).

**Results:**

Symptom severity was negatively correlated with QoL (*r* = −0.69, *P* < 0.01) and active acceptance (*r* = −0.47, *P* < 0.01), and positively correlated with negative acceptance (*r* = 0.56, *P* < 0.01). Active acceptance was positively (*r* = 0.60) and negative acceptance negatively (*r* = −0.60) correlated with QoL (both *P* < 0.01). Mediation analysis revealed two significant indirect pathways: symptoms → reduced active acceptance → lower QoL, accounting for 23.92% of total effect (95% bootstrap CI [−0.068, −0.033]), and symptoms → increased negative acceptance → lower QoL, accounting for 25.36% of total effect (95% bootstrap CI [−0.074, −0.035]). Neither confidence interval contained zero.

**Conclusion:**

Both active acceptance and negative acceptance independently mediate the relationship between physical symptoms and QoL in patients with GICs. Active acceptance buffers the impact of symptoms, whereas negative acceptance exacerbates it. These findings support integrating psychological interventions that foster active acceptance and mitigate negative acceptance as a core component of symptom management to optimize QoL in this population.

## Introduction

The global incidence of cancer is projected to increase by 69% annually by 2030 [1], affecting millions of people worldwide each year. Accounting for 21% of all deaths in both men and women, cancer represents the second leading cause of mortality, surpassed only by heart disease [2]. The incidence of gastrointestinal cancers (GICs) includes mainly colorectal cancer (9.4%), liver cancer (8.3%), gastric cancer (7.7%), pancreatic cancer (4.7%), and bile duct cancer (0.3%–0.5%) [3]. As the incidence of GICs continues to increase, then have become a major cause of cancer-related mortality, second only to lung cancer [4]. Concurrent with advancements in oncological therapeutics, patient survival durations are progressively increasing [5]. This paradigm shift necessitates a heightened focus on patient quality of life (QoL) beyond merely extending lifespan. In recent years, optimizing QoL has emerged as a critical priority for healthcare professionals, patients, and their families.

QoL comprises two fundamental dimensions: the capacity to perform physical and psychological activities of daily living and the patient’s perceived satisfaction with their functional status and disease management [6]. For patients with GICs, physical symptom burden is the most significant determinant of QoL [7]. Compared with patients with other cancer types, GIC patients frequently endure a more substantial symptom burden. Pancreatic cancer often manifests severe pain; colorectal cancer precipitates bloating, nausea, and diarrhea and frequently necessitates ostomy creation postoperatively [8]; and gastric cancer may lead to profound malnutrition and cachexia, compounded by pervasive symptoms such as fatigue and insomnia [9, 10]. Furthermore, adverse effects from anti-neoplastic therapies frequently exacerbate this symptom complex [11], collectively contributing to substantial deterioration in QoL. For example, long-term use of painkillers can lead to constipation; patients experiencing multiple episodes of diarrhea daily may become confined to areas near restrooms, whereas those with ostomies might even be unable to participate in normal social activities in public settings. Patients’ physical and psychological symptoms directly impact their QoL. While there is extensive research on the relationship between symptoms and QoL across various cancer types, the mediating pathways and mechanisms underlying this relationship have received limited exploration.

Illness acceptance attitudes encompass patients’ cognitive integration and emotional adaptation to their disease, potentially modulating their symptom experience through coping strategy modification. Research has dichotomized disease acceptance into acceptance and nonacceptance. Illness acceptance denotes adaptation to stressful reality, learning to coexist with the condition, and acknowledging its impact and irreversible trajectory [12], thereby theoretically exerting a positive influence on QoL. Nonacceptance or denial of an illness, in contrast to acceptance, is an unconscious defensive attitude adopted to cope with overwhelming psychological distress [13]. This stance prevents patients from adopting positive approaches to mitigate the harm of the illness, thereby negatively impacting their QoL. Paradoxically, some evidence suggests that illness acceptance may also negatively impact QoL. This apparent contradiction stems from frequent oversimplification of “acceptance” as a uniformly positive combatant attitude, neglecting its multidimensional complexity—particularly the crucial distinction between active and negative acceptance of illness [14, 15]. Active acceptance of illness involves constructive engagement with the disease, proactive problem solving, and developing competencies for illness management [16]. Active acceptance of illness influences QoL primarily by enabling patients to increase their overall life satisfaction through positive psychological adjustment and behavioral changes. Research indicates that active acceptance of illness is significantly associated with better treatment adherence and a higher QoL [17, 18]. In contrast, negative acceptance of illness, or “resignation,” implies an acknowledgment of reality that is coupled with a perceived inability to change one’s external circumstances. This state is often characterized by a relinquishment of active coping efforts and negative expectations for the future, thereby exerting a negative effect on the patient’s QoL [19]. Patients exhibiting negative acceptance typically demonstrate maladaptive compliance, deficient proactive coping, negative therapeutic attitudes, and potential treatment abandonment, collectively substantially compromising QoL [20].

Current research predominantly examines direct associations between illness acceptance attitudes and QoL, with insufficient investigation of mediating pathways through physical symptoms.

This study hypothesizes that illness acceptance attitudes indirectly enhance QoL through physical symptom attenuation (mediation model: illness acceptance attitudes → physical symptoms → QoL).

Accordingly, we designed a cross-sectional investigation targeting patients with GICs (including those with gastric cancer, colorectal cancer, hepatic cancer, pancreatic cancer, and biliary duct cancer). Using standardized instruments, we assessed physical symptom severity, illness acceptance attitudes (differentiating active and negative dimensions), and QoL to examine their interrelationships and investigate the mediating effects of acceptance types in the symptom‒QoL pathway. The ultimate objective is to ensure that, beyond effective pharmacological symptom control, patients achieve psychological well-being, adopt constructive illness acceptance attitudes, and concurrently enhance their QoL.

## Methods

### Study Design

Participants were recruited from the Cancer Center of Union Hospital, Tongji Medical College, Huazhong University of Science and Technology, from April to October 2024, and informed consent was obtained from all participants or their authorized family members. Prior to commencing formal data collection, all the researchers received uniform training to ensure consistent interpretation of the questionnaires. Researchers explained the purpose and significance of the study to patients who met the inclusion criteria and conducted the survey via standardized questionnaires. Completing the questionnaires requires approximately 30–40 min. During the data collection process, researchers addressed any incomplete items, errors, or multiple selections by asking patients for clarification and recording the corrections. Patients retained the right to withdraw from the study at any time without penalty. All the information collected in this study adhered strictly to the principles of confidentiality.

### Study Subjects

A convenience sampling method was employed to select patients of different ages, sexes, educational levels, and diagnoses.

The inclusion criteria were as follows: (1) diagnosis of GICs by a doctor; (2) aware of the cancer diagnosis; (3) estimated survival for more than three months; and (4) aged 18 years or older.

The exclusion criteria were as follows: (1) being unaware of a cancer diagnosis; (2) being diagnosed with psychiatric disorders or cognitive impairment; and (3) having comorbid chronic diseases.

### Measurement Tools

#### General Data Survey

The questionnaire was compiled by combining four scales selected from the literature review as suitable for this study. Sociodemographic data primarily included sex, age, educational level, occupation, place of residence, marital status, and disease duration. The assessment tools included the MDASI, SF-12, ICQA, and MCMQ.

#### MDASI

The MDASI is a well-validated scale for assessing symptom burden in cancer patients [21]. It includes 13 items assessing the severity of the most common cancer-related symptoms (e.g., pain, fatigue, nausea). Each item was rated on an 11-point Likert scale ranging from “0 = not present” to “10 = as bad as you can imagine”. The reliability of the scale was satisfactory, with a Cronbach’s alpha coefficient of 0.90 in the present study. A score of 1–4 indicates mild symptoms, a score of 5–6 indicates moderate symptoms, and a score of 7 or above is considered severe.

#### SF-12

The SF-12, developed by John E. Ware et al., is a shortened version of the SF-36 Health Survey designed to assess health-related QoL efficiently. It contains 12 items covering two dimensions: physical and mental health. Weighted regression was used to calculate the physical component summary (PCS-12) and mental component summary (MCS-12) scores. The scale scores range from 0 to 100, with higher scores indicating better health status in that dimension. A score above 50 is generally considered indicative of better overall QoL, whereas a score below 50 suggests poorer overall QoL. A score of 50 points indicates an average level and serves as a benchmark for evaluation [22]. The SF-12 has high validity and reliability, explains more than 90% of the variance in the SF-36, has been translated into 30 languages, and is suitable for large-scale health monitoring and international studies [23].

#### ICQA

The ICQA, developed by Evers et al., was used to assess patients’ active acceptance. This questionnaire consists of three subscales: helplessness, acceptance, and perceived benefits. It employs a 4-point Likert scale (1 = totally disagree, 4 = totally agree). Each item is scored from 1 to 4, yielding a total score ranging from 6 to 24 [24]. A higher total score indicates a stronger level of active acceptance. Scores ≤ 12 represent low active acceptance, scores between 13 and 18 represent moderate active acceptance, and scores ≥ 19 represent high active acceptance.

#### MCMQ

The MCMQ, developed by Feifel H, was used to assess patients’ negative acceptance [25]. It uses a 4-point continuous rating scale for responses (e.g., from “never” to “always”, “very little” to “very much”). Each item is scored from 1 to 4, resulting in a total score ranging from 5 to 20 [26]. A higher total score indicates a stronger tendency toward negative acceptance. Scores of 5–9 indicate low negative acceptance, scores of 10–14 indicate moderate negative acceptance, and scores of 15–20 indicate high negative acceptance.

### Statistical Analysis

Data analysis in this study was performed via SPSS 29.0. First, descriptive statistical analysis was conducted on the study data. Second, the reliability and validity of the four scales (MDASI, SF-12, ICQA, MCMQ) were tested. Internal consistency was assessed via Cronbach’s α coefficient, with α ≥ 0.70 considered indicative of acceptable reliability. Validity was considered acceptable with a KMO measure ≥ 0.60. Third, Pearson correlation analysis was used to examine the relationships between physical symptoms, active acceptance attitudes, negative acceptance attitudes, and QoL. A *P* value < 0.01 was considered statistically significant. Fourth, the impact of physical symptoms on QoL was analyzed via logistic regression, with a *P* value < 0.01 indicating statistical significance. Fifth, a structural equation modeling approach (SPSS PROCESS, Model 4) was employed to test the mediating roles of active acceptance attitudes and negative acceptance attitudes in the relationship between symptoms and QoL. The bootstrap sampling method (with 5000 iterations) was used, and 95% confidence intervals (CIs) were calculated. A *P* value < 0.001 was considered statistically significant for the mediation analysis. Furthermore, we confirmed that the key assumptions for the regression and mediation analyses were tested and met, including checks for multicollinearity (with all the VIFs < 5) as well as the normality and independence of the residuals.

## Results

### Analysis of Demographic and Clinical Characteristics of Patients with GICs

A total of 318 questionnaires were distributed (278 electronic versions and 40 paper versions). A total of 17 questionnaires were invalid, resulting in 301 valid questionnaires and a response rate of 94.65%. The study subjects consisted of 301 inpatient and outpatient patients with GICs. Among them, 63.46% were male, and 36.54% were female, with ages ranging from 23 to 78 years. Patients aged > 50 years accounted for 77.41% of the sample. The distribution of cancer types was as follows: gastric cancer, 19.27%; pancreatic cancer, 15.28%; colorectal cancer, 50.17%; liver cancer, 9.63%; bile duct cancer, 3.99%; and other, 1.66% (gastrointestinal stromal tumors and ampullary carcinoma). According to statistics, 65.45% of the patients had a history of previous surgery. Newly diagnosed patients accounted for 14.95% of the sample, whereas follow-up patients accounted for 85.05%. The proportions of patients who underwent chemotherapy, immunotherapy, targeted therapy, radiotherapy and combination therapy (using two or more treatment modalities, such as chemotherapy combined with immunotherapy or chemotherapy combined with targeted therapy) were 18.93%, 4.65%, 8.31%, 1.33% and 64.79%, respectively. Patients with recurrent disease accounted for 62.79% of the cohort, whereas those receiving adjuvant therapy constituted 37.21%. Among all patients, 19.27%, 10.30%, 58.47%, and 11.96% had stage I, II, III, and IV disease, respectively (Table 1).

**Table 1:** Summary of characteristics of patients

Characteristics	*n*	Percentage (%)
Gender		
Male	191	63.46
Female	110	36.54
Marital status		
Married	290	96.34
Single	7	2.33
Divorced	4	1.33
Age		
≤ 50 years old	68	22.59
> 50 years old	233	77.41
Place of residence		
Urban	125	41.53
Rural	176	58.47
Ethnicity		
Han ethnicity	294	97.67
Minority ethnicity	7	2.33
Types of tumors		
Gastric cancer	58	19.27
Pancreatic cancer	46	15.28
Colorectal cancer	151	50.17
Liver cancer	29	9.63
Cholangiocarcinoma	12	3.99
Others	5	1.66
Patient types		
First-time patients	45	14.95
Follow-up patients	256	85.05
Treatment methods		
Chemotherapy	57	18.93
Immunotherapy	14	4.65
Targeted therapy	25	8.31
Radiotherapy	4	1.33
Combination therapy	195	64.79
Others	6	1.99
Surgery (previous)	198	65.45
Cancer recurrence or progression		
Yes	189	62.79
No	112	37.21
Cancer stage		
I	58	19.27
II	31	10.30
III	176	58.47
IV	36	11.96

### Reliability and Validity Testing of the Four Scales

Reliability and validity tests were conducted for the four scales: the MDASI, SF-12, ICQA, and MCMQ. The Cronbach’s α coefficients for all scales exceeded 0.70 (MDASI: α = 0.903; SF-12: α = 0.826; ICQA: α = 0.825; MCMQ: α = 0.865), indicating good internal consistency. The KMO measures for validity were all greater than 0.60 (MDASI: KMO = 0.896; SF-12: KMO = 0.844; ICQA: KMO = 0.900; MCMQ: KMO = 0.872), demonstrating satisfactory validity. These results confirm that the measurement tools used in this study are reliable and credible (Table 2) and are suitable for confirmatory factor analysis.

**Table 2:** Reliability and validity test results of physical symptoms, quality of life, and illness acceptance attitude scales

Scales	Cronbach’s α	Model fit indices (*χ*^2^/df, RMSEA, CFI)
MDASI-GI	0.903	6.683, 0.138, 0.807
SF-12	0.826	5.978, 0.129, 0.894
ICQA	0.825	10.14, 0.175, 0.957
MCMQ	0.865	4.896, 0.114, 0.979

### Relationships Among Active Acceptance Attitudes, Negative Acceptance Attitudes, Physical Symptoms, and QoL

Pearson correlation analysis revealed that physical symptoms had a significant negative effect on QoL (*r* = −0.69, *P* < 0.01). Physical symptoms had a significant negative effect on active acceptance attitudes (*r* = −0.47, *P* < 0.01) and a significant positive effect on negative acceptance attitudes (*r* = 0.56, *P* < 0.01). Furthermore, active acceptance had a significant positive effect on QoL (*r* = 0.60, *P* < 0.01), whereas negative acceptance had a significant negative effect on QoL (*r* = −0.60, *P* < 0.01) (Table 3).

**Table 3:** Validity test results of physical symptoms, quality of life, and illness acceptance attitude scales

Scales	KMO	Degrees of freedom	Bartlett’s test of sphericity	*P* value
Physical symptoms	0.896	78	1962.115	< 0.01
Quality of life	0.844	36	1058.197	< 0.01
Active acceptance attitude	0.900	15	1931.328	< 0.01
Negative acceptance attitude	0.872	10	943.361	< 0.01

### Mediating Role of Active and Negative Acceptance Attitudes in Relationship between Physical Symptoms and QoL

An active acceptance attitude was significantly associated with a reduction in physical symptom levels (*β* = −0.16, *P* < 0.0001), which in turn contributed to an improved QoL (*β* = −0.05, *P* < 0.0001). The total effect for this pathway was −0.209 (95% CI: [−0.697, −0.184]). A significant mediating effect of active acceptance attitudes was observed (*β* = −0.050, SE = 0.009, 95% CI: [−0.068, −0.033]), accounting for 23.92% of the total effect. These results indicate that an active acceptance attitude enhances QoL by alleviating physical symptoms (Table 4).

**Table 4:** Mediating effect of active acceptance attitudes

Paths	Effect size	SE	95%CI	Proportion of total effect
Total effect (Physical symptoms → Quality of life)	−0.209	0.012	(−0.697, −0.184)	
Direct effect (Controlling for active acceptance attitudes)	−0.159	0.013	(−0.533, −0.134)	76.08%
Indirect effect (Mediated by active acceptance attitudes)	−0.050	0.009	(−0.068, −0.033)	23.92%

The mediating effect of negative acceptance attitudes was slightly weaker (*β* = −0.053, SE = 0.009, 95% CI: [−0.074, −0.035]), accounting for 25.36% of the total effect. These findings suggest that a negative acceptance attitude also indirectly improves QoL by alleviating physical symptoms (Table 5).

**Table 5:** Mediating effect of negative acceptance attitudes

Paths	Effect size	SE	95%CI	Proportion of total effect
Total effect (Physical symptoms → Quality of life)	−0.209	0.012	(−0.697, −0.184)	
Direct effect (Controlling for negative acceptance attitudes)	−0.156	0.014	(−0.529, −0.129)	74.64%
Indirect effect (Mediated by negative acceptance attitudes)	−0.053	0.1	(−0.074, −0.035)	25.36%

**Fig. 1:**
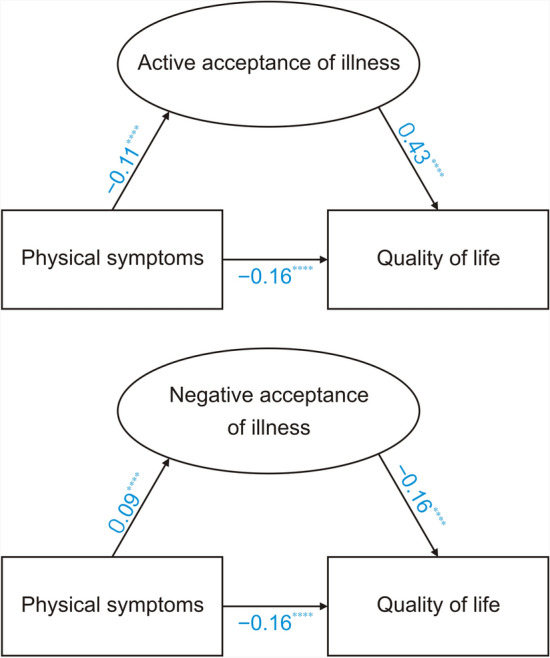
Mediation results diagram. ^****^P < 0.0001

As illustrated in Fig. 1, the results demonstrate that (1) both active and negative acceptance attitudes indirectly improved QoL through the reduction of physical symptoms (*P* < 0.0001); (2) In the survey, we observed that the proportion of the indirect effect for negative acceptance attitudes (25.36%) was marginally greater than that for active acceptance attitudes (23.92%) among patients with GICs; and (3) the 95% CIs for both pathways did not include 0, supporting the statistical robustness of the mediating effects.

## Discussion

This study revealed significant interactions among disease acceptance attitudes, physical symptoms, and QoL in patients with GICs. Patients with more severe physical symptoms tend to report poorer QoL [27] and are less inclined to adopt an attitude of active acceptance [28]. Conversely, those who demonstrated active acceptance generally experienced a higher QoL. In contrast, patients who were subjectively employed with negative acceptance showed a marked decline in their QoL [29]. This research is the first to elucidate the relationships and underlying mediating mechanisms among these three dimensions—disease acceptance attitudes, physical symptoms, and QoL—in patients with GICs from a comprehensive, tripartite perspective. The findings can guide healthcare professionals in more accurately identifying whether a patient possesses an active or negative acceptance attitude and understanding the subsequent impact on QoL. This knowledge is crucial for developing and implementing more effective and targeted interventions. The ultimate goal is to increase the efficacy of pharmaceutical treatments by encouraging patients to face their disease proactively, ameliorate their symptoms, and thereby maximize the overall effectiveness of their treatment regimens. Furthermore, if patients learn to actively accept their condition, it may reduce cancer-related stigma, foster a more positive attitude toward the illness, and strengthen their courage to combat the disease. This psychological shift can alleviate symptom burden and enhance QoL.

This study reveals distinct psychological pathways through which disease acceptance attitudes affect QoL via physical symptoms. Active acceptance significantly mediated 23.92% of the total effect, demonstrating its protective role in enhancing QoL through symptom reduction. Negative acceptance had a slightly weaker mediating effect (*β* = −0.053, 95% CI [−0.074, −0.035]), accounting for 25.36% of the total effect, indicating its indirect role in QoL improvement through symptom alleviation. From a theoretical perspective, we posit that active acceptance—an adaptive coping strategy—buffers the impact of symptoms by enhancing self-efficacy (e.g., through proactive stoma management or optimized treatment adherence), thereby forming a protective pathway: “Worsening symptoms → Decreased active acceptance → Deteriorated QoL”. Conversely, negative acceptance—a form of resigned acquiescence—triggers a vicious cycle of “learned helplessness-behavioral withdrawal” (e.g., social avoidance, treatment abandonment), leading to a detrimental pathway: “Worsening symptoms → Increased negative acceptance → Deteriorated QoL”. The dual-pathway model proposed in this study poses a significant challenge to traditional research that views acceptance as a unified adaptive concept: while passive acceptance may alleviate internal conflicts in the short term, it harms long-term quality of life through a vicious cycle of behavioral withdrawal. This directly contradicts the oversimplified assumption that “acceptance is always beneficial”. Simultaneously, this model aligns with the emerging multidimensional theory of acceptance, indicating that active acceptance aligns with the notion of positive adaptation in traditional research, whereas passive acceptance is closer to concepts such as “resignation” or “surrender”. This not only drives theoretical and clinical practices toward a more refined differentiation but also provides a theoretical basis for developing targeted clinical interventions on the basis of patients’ acceptance attitudes.

Furthermore, the comparable mediation proportions of both active (23.92%) and negative (25.36%) acceptance have significant clinical implications. These findings indicate that nearly one quarter of symptom-related impairments in QoL are potentially modifiable through psychological interventions.

Specific translational directions include the following: (1) Adjusting intervention focus: The stronger effect size of the active acceptance path (*β*-active = −0.16 vs. *β*-negative = −0.12) suggests prioritizing the cultivation of active coping skills. For patients with low active acceptance (e.g., ICQA score ≤ 12), employing acceptance and commitment therapy (ACT) to deliver “cognitive defusion-value-guided behavior” training—such as reframing “living with cancer” to “life restructuring”—can significantly increase self-efficacy in managing modifiable symptoms such as bowel dysfunction or malnutrition [30, 31]. (2) Blocking the high-risk pathway: For the negative acceptance high-risk group (e.g., MCMQ score ≥ 15, often observed in elderly or advanced-stage patients), interventions must target the “symptoms → negative acceptance” link via cognitive behavioral therapy (CBT) [32, 33]. By challenging catastrophic beliefs (e.g., “pain means the end of life”) and incorporating graded social exposure exercises, the cycle of behavioral withdrawal can be effectively disrupted. (3) Symptom‒psychology-matched intervention: For high-distress symptoms (e.g., pancreatic cancer pain), pharmacological analgesia can be combined with mindfulness-based pain reduction techniques to block the negative acceptance pathway [34]. For functional impairment symptoms (e.g., gastric cancer cachexia), the active acceptance pathway can be activated through nutritional support coupled with empowerment education, such as providing self-management symptom monitoring manuals.

Following the identification of the mediating relationships among acceptance of illness, physical symptoms, and QoL in patients with GICs, we constructed an integrated “Psychological‒Physical‒Functional” triad intervention framework. Acceptance of illness influences symptom experience and functional outcomes through both psychological and behavioral pathways [35, 36]. At the psychological level, active acceptance of illness facilitates cognitive restructuring, helping patients reframe their illness as a manageable challenge, thereby significantly buffering the impact of symptoms [36]. In contrast, negative acceptance of illness triggers emotional suppression, accelerating a vicious cycle of despair and diminishing coping capacity [37]. Behaviorally, individuals with active acceptance are more inclined toward proactive symptom management and maintaining social participation, whereas those with negative acceptance are prone to treatment abandonment and social avoidance [38]. This dual “psychological‒behavioral” mechanism ultimately affects QoL through the mediating effect of physical symptoms: our findings indicate that approximately one-quarter of symptom-related impairments in QoL are attributable to acceptance attitudes, with the protective effect of active acceptance being particularly prominent [39, 40]. These findings suggest that clinical interventions must extend beyond conventional symptom control and target psychological restructuring as a core strategy for improving functional outcomes. This study is the first to validate the dual pathway mediating the effect of attitudes toward acceptance of illness in a GIC population. Grounded in the integrated “Psychological-Physical-Functional” perspective, we interpret the clinical implications of our findings across three dimensions.

### Psychological Dimension: Acceptance Attitude toward Illness as the Core Hub for Symptom Cognition Transformation

The acceptance of illness serves as a central variable in psychological regulation, directly determining how patients cognitively process physical symptoms [41]. This study revealed that active acceptance of illness enhances cognitive restructuring—reframing the illness as a manageable challenge rather than a hopeless situation—thereby significantly buffering the psychological impact of symptoms. In contrast, negative acceptance of illness triggers a learned helplessness mechanism, trapping patients in a vicious cycle of “worsening symptoms → deepening despair → coping abandonment”. In clinical practice, this underscores the necessity of incorporating acceptance of attitude screening into routine psychological assessments. For patients with high negative acceptance tendencies (particularly those with MDASI scores ≥ 7), priority should be given to using ACT to break cognitive rigidity and foster psychological resilience for “living with the illness”. For those with low active acceptance, meaning-centered group psychotherapy (MCGP) can be implemented to strengthen the integrative capacity for illness, thereby establishing a psychological foundation for managing physical symptoms.

### Physical Dimension: Interactive Mechanism between Symptom Characteristics and Psychological Responses

Physical symptoms do not exist in isolation; their type and intensity exert differential health effects through psychological mediation.

High-distress symptoms, such as pancreatic cancer pain and chemotherapy-induced vomiting, directly activate the negative acceptance pathway, where intense physiological discomfort readily triggers a sense of loss of control. These cases require combined pharmacological-psychological interventions, such as opioids coupled with mindfulness-based pain management training. In contrast, functional impairment symptoms, including stoma-related issues in patients with colorectal cancer and cachexia in patients with gastric cancer, are more susceptible to modulation by active acceptance of illness, as they can be improved through skill-based training, such as stoma care education, making them suitable for integrated rehabilitation and cognitive behavioral programs. Notably, negative acceptance of illness may exacerbate physical symptoms such as fatigue and insomnia through mechanisms such as HPA axis activation and sustained cortisol elevation, forming a negative “psychological-physiological” feedback loop. Therefore, in clinical practice, differentiated symptom management pathways should be established: for refractory symptoms such as neuropathic pain, neuromodulation should be combined with cognitive defusion techniques; for functional symptoms such as nutritional disorders, self-monitoring and adaptive behavior training should be enhanced.

### Functional Dimension: The Terminal Pathway of Social Functional Reconstruction Driven by Acceptance of Illness

Functional recovery serves as a core indicator of QoL, and disease acceptance of illness directly influences functional outcomes through behavioral adaptation [42]. Patients with active acceptance of illness are more likely to maintain treatment adherence, such as tolerating side effects to complete chemotherapy, and preserve social role functioning, including returning to work, leading to a higher QoL. In contrast, those with negative acceptance of illness often exhibit behavioral withdrawal, such as avoiding stoma which resulting in social isolation and activity limitations, which accelerate functional decompensation and ultimately diminish QoL.

The clinical implication lies in establishing a function-oriented, three-stage intervention chain: (1) Short-term (patient phase): This chain aims to block negative acceptance by providing crisis-related psychological intervention for patients with high symptom burden (e.g., MDASI score ≥ 5). (2) Medium-term (rehabilitation phase): Fostering active acceptance through empowerment education (e.g., self-management symptom manuals) to promote the recovery of daily living activities. (3) Long-term (survivorship phase): Rebuild social participation (e.g., social skills training for patients with stomas) through peer support and vocational rehabilitation programs, ultimately achieving comprehensive biopsychosocial functional integration.

### Study Limitations and Future Directions

This study has several limitations. First, the single-center design and limited sample size may restrict the generalizability of our findings. Second, the analysis of standardized scales remains basic and lacks an exploration of the mechanisms underlying factor interactions. Third, as a cross-sectional study, it cannot establish causality or capture the evolution of psychological states over the cancer trajectory. While the mediation model is theoretically sound, the observed associations could be bidirectional; longitudinal studies are needed to confirm directionality. Fourth, the absence of systematically collected clinical data (e.g., Eastern Cooperative Oncology Group [ECOG] performance status) limits fuller contextual interpretation.

These limitations indicate that future research should employ longitudinal designs to analyze and validate the identified factors over an extended period. Furthermore, expanding the study to include multiple centers and larger, more diverse samples would increase the generalizability of the findings. The use of advanced analytical methods to explore the interaction mechanisms among factors could provide deeper insights into the relationships among symptoms, QoL, and acceptance of illness in cancer patients. By addressing these limitations, future studies can build upon our findings to provide more robust and generalizable evidence regarding the psychological distress experienced by patients with GICs.

## Conclusion

Significant correlations exist among physical symptoms, illness attitudes, and QoL in patients with GICs. Furthermore, patients’ acceptance of illness plays a crucial mediating role in the impact of physical symptoms on QoL. Consequently, the relationships among a patient’s physical symptoms, acceptance of illness, and QoL are complex and intricately intertwined. This study underscores the importance of not only exploring cancer treatments but also proactively addressing whether a patient’s acceptance of illness is conducive to their overall care. The ultimate goal is to achieve effective symptom control and enhance patients’ QoL.

## Data Availability

The datasets generated and analyzed during the current study are not publicly available due to patient privacy and ethical restrictions but are available from the corresponding author upon reasonable request.
